# A prediction model for permanent pacemaker implantation after transcatheter aortic valve replacement

**DOI:** 10.1186/s40001-023-01237-w

**Published:** 2023-07-29

**Authors:** Yiming Qi, Xiaolei Lin, Wenzhi Pan, Xiaochun Zhang, Yuefan Ding, Shasha Chen, Lei Zhang, Daxin Zhou, Junbo Ge

**Affiliations:** 1 Department of Cardiology, Zhongshan Hospital, Shanghai Institute of Cardiovascular Diseases, Fudan University, 180 Fenglin Road, Shanghai, 200032 China; 2 National Clinical Research Center for Interventional Medicine, Shanghai, China; 3 School of Data Science, Fudan University, Shanghai, China

**Keywords:** TAVR, Pacemaker implantation, Heart block, Risk prediction, Mechanical stress

## Abstract

**Background:**

This study aims to develop a post-procedural risk prediction model for permanent pacemaker implantation (PPMI) in patients treated with transcatheter aortic valve replacement (TAVR).

**Methods:**

336 patients undergoing TAVR at a single institution were included for model derivation. For primary analysis, multivariate logistic regression model was used to evaluate predictors and a risk score system was devised based on the prediction model. For secondary analysis, a Cox proportion hazard model was performed to assess characteristics associated with the time from TAVR to PPMI. The model was validated internally via bootstrap and externally using an independent cohort.

**Results:**

48 (14.3%) patients in the derivation set had PPMI after TAVR. Prior right bundle branch block (RBBB, OR: 10.46; *p* < 0.001), pre-procedural aortic valve area (AVA, OR: 1.41; *p* = 0.004) and post- to pre-procedural AVA ratio (OR: 1.72; *p* = 0.043) were identified as independent predictors for PPMI. AUC was 0.7 and 0.71 in the derivation and external validation set. Prior RBBB (HR: 5.07; *p* < 0.001), pre-procedural AVA (HR: 1.33; *p* = 0.001), post-procedural AVA to prosthetic nominal area ratio (HR: 0.02; *p* = 0.039) and post- to pre-procedural troponin-T difference (HR: 1.72; *p* = 0.017) are independently associated with time to PPMI.

**Conclusions:**

The post-procedural prediction model achieved high discriminative power and accuracy for PPMI. The risk score system was constructed and validated, providing an accessible tool in clinical setting regarding the Chinese population.

**Supplementary Information:**

The online version contains supplementary material available at 10.1186/s40001-023-01237-w.

## Background

Transcatheter aortic valve replacement (TAVR) is a safe and effective treatment for patients with severe aortic stenosis at even low surgical risk [1, 2]. Permanent pacemaker implantation (PPMI) remains a common complication after TAVR compared to surgical aortic valve replacement (SAVR) [3, 4]. In a recent systematic review, 22.7% of patients developed permanent left bundle branch block (LBBB) after TAVR, and 5.9% to 32.0% required PPMI [5].

PPMI after TAVR not only increases the occurrence of wire-related tricuspid regurgitation, implant infection, wire damage, pocket damage and hematoma, cardiac perforation and other complications [6, 7], but also increases the economic burden of patients and the length of hospital stay [8]. The results of a recent meta-analysis further confirmed a significant increase in mortality among patients requiring PPMI after TAVR [5].

Previous studies suggested that prior right bundle branch block (RBBB) [9, 10], short membranous septum length [11, 12], calcification of device landing zone [10, 13], intra-procedural atrioventricular block (AVB) [4], prosthesis oversizing [14], self-expanding valve [15, 16], and lower implantation depth [10, 13] were associated with PPMI after TAVR, but no single factor could predict PPMI accurately.

The majority of the existing prediction models for PPMI only used pre-procedural predictors [17–19]. However, for post-procedural patients, there still lacks effective prediction models for PPMI risk assessment and timely guidance of pacemaker removal and patient discharge. In this study, we aim to develop a post-procedural prediction model for patients requiring PPMI after TAVR, on top of the pre-procedural risk prediction model.

## Methods

### Study population

This study was approved by Ethic Committee of ZhongShan Hospital, Fudan University (NO. B2020-039), and written informed consents were obtained. A total of 381 patients with aortic stenosis were treated with TAVR at Zhongshan Hospital (a tertiary teaching hospital affiliated to Fudan University) in Shanghai, China from June 2015 to September 2021. After excluding patients with previous PPMI, previous TAVR or SAVR, concurrent percutaneous coronary intervention (PCI) or septal myocardial ablation (SMA), electric cardioversion during procedure, or transferring to surgical operation due to complications and death, 336 patients were included in the analysis. For external validation, 48 patients from the same hospital were included from October 2021 to February 2022 according to the same including and excluding criteria.

### Data acquisition, pre-processing and endpoint definition

Baseline characteristics, medical history, pre-procedural electrocardiography (ECG), procedural characteristics, peri-procedural transesophageal/transthoracic echocardiography (TEE/TTE) and laboratory results were acquired for each patient. Aortic valve area (AVA) and pressure gradient were extracted from TEE or TTE when TEE was missing for that patient. Troponin-T (TnT) was measured within 3 days before and 1 day after TAVR. Prosthetic nominal area (PNA) was calculated according to the diameter of the valve. Percent oversizing of prosthetic valve was calculated as a ratio of nominal valve perimeter after the procedure divided by measured aortic annulus perimeter on computed tomography. In addition to these characteristics, post- and pre-procedural difference of TnT (△TnT), post- to pre-procedural ratio of AVA (AVA ratio) and post-procedural AVA to PNA ratio (AVA–PNA ratio) were also included to reflect the post-procedural changes relative to the pre-procedural condition. The primary endpoint was whether patients received PPMI within 60 days of TAVR due to complete heart block (CHB), high-degree atrioventricular block (HAVB), sinus arrest or symptomatic bradycardia. The secondary endpoint was the time-to-PPMI, defined as the days between TAVR and PPMI for patients with PPMI, and censored for those without PPMI. The need for PPMI was evaluated by a consensus committee consisting of experienced cardiac electrophysiology specialist and interventional cardiologists. The postoperative length of stay (PLOS) was defined as days from the date of TAVR to discharge.

### TAVR procedure

The decision to undergo TAVR was made by our heart team including interventional cardiologists and cardiac surgeon according to Chinese guidelines and consensus [20, 21]. The type, sizing, access, balloon pre- and post-dilation were determined by the clinical consensus team, including experienced clinical and interventional cardiologists. The self-expandable valves we used including Venus-A (Venus Medtech, Hangzhou, China) [22] and VitaFlow (MicroPort, Shanghai, China) [23]. The balloon-expandable valve we used is Sapien3 (Edwards Lifesciences, Irvine, CA, USA).

### Statistical analysis

Continuous characteristics were described using mean and standard deviation, while categorical characteristics were presented using frequency and proportion per category. *p* values for comparing patient characteristics were obtained using two-sample *T* test for continuous predictors and Chi-square or Fisher’s exact test for categorical predictors. Missingness was generally low (below 15%, Additional file 1: Table S3) for all predictors and multiple imputation was used to impute missing values. Univariate logistic regression was performed for each predictor and those with significant *p* values were selected as candidate predictors. A systematic review of risk factors for post-procedural PPMI in TAVR patients [11, 17, 18, 24] were also used to select candidate predictors. Expert opinions were adopted when necessary to confirm relevant predictors or include predictors with large *p* values but potentially clinical significance. To develop pre- and post-procedural prediction models, forward and backward variable selection was first performed to select significant pre-procedural predictors for PPMI. The selected pre-procedural predictors were then included in the forward and backward variable selection process together with the post-procedural characteristics to yield significant pre- and post-procedural predictors. Multivariate logistic regression model was used to associate the selected pre- and post-procedural predictors with the risk of PPMI. Area under the receiver operating characteristic curve (AUROC) was calculated to evaluate the diagnostic and discriminative performances of the predictive models. *p* values for comparing two AUROCs were obtained using non-parametric Mann–Whitney statistical test. Individual risk scores were calculated for each predictor value based on the estimated probabilities of PPMI from the multivariate logistic regression model, and total risk score (on the scale of 0 to 100) for each participant was obtained by adding up the individual risk scores associated with predictor values associated with that participant. Time-to-event analysis using Cox proportional hazard model was also performed to identify important predictors to explain how soon PPMI was required among patients who underwent TAVR. Internal validation was conducted via bootstrap. Specifically, 5000 bootstrap samples were obtained from the original patients, the pre- and post-procedural prediction models were applied, and AUROCs were calculated and averaged to examine the internal validity of the predictive models. External validation was conducted using an independent cohort consisting of 48 patients from the same hospital. All statistical analyses were performed using R version 4.1.2.

## Results

### Patient characteristics

**Fig. 1:**
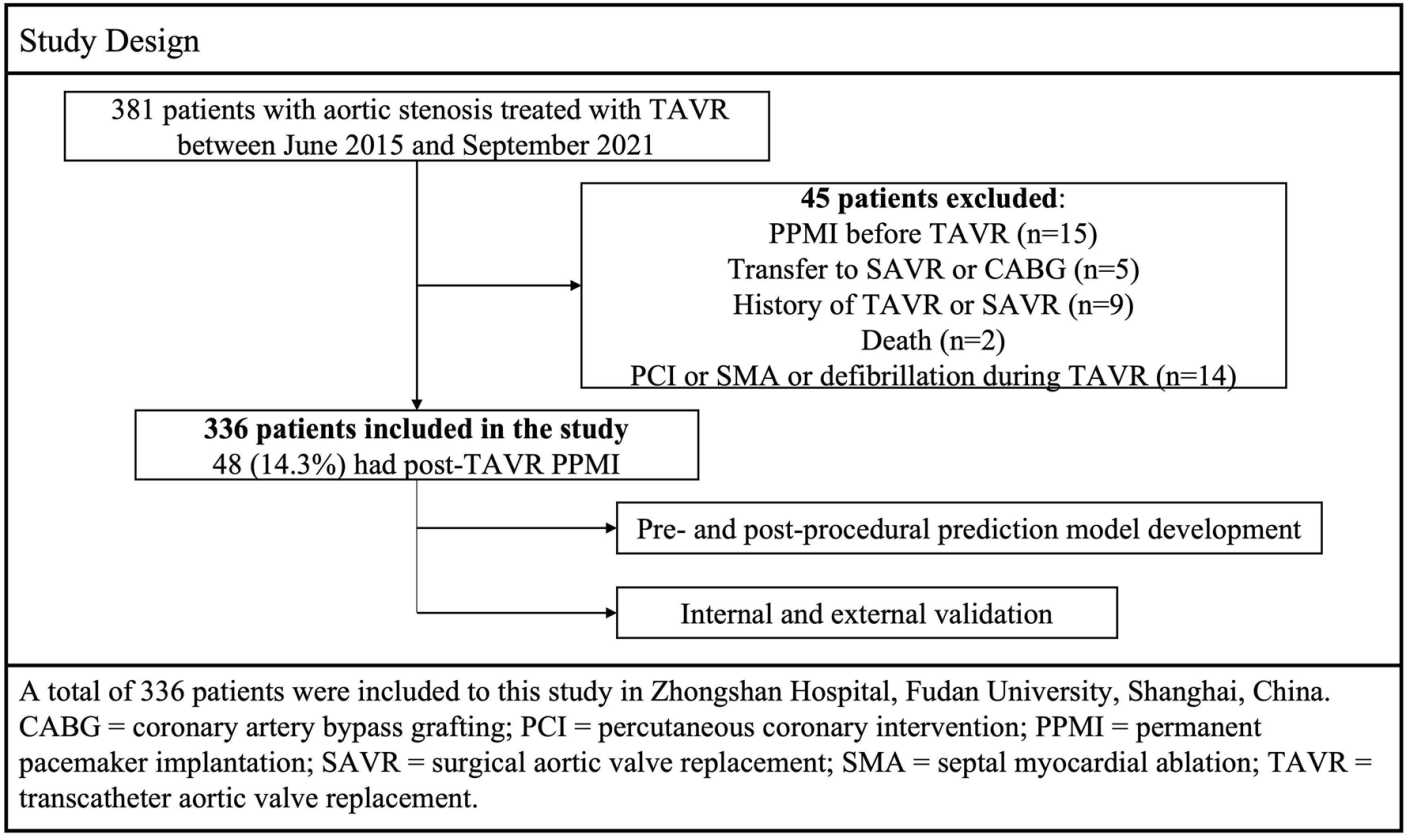
Study design. A total of 336 patients were included to this study in Zhongshan Hospital, Fudan University, Shanghai, China. CABG, coronary artery bypass grafting; PCI, percutaneous coronary intervention; PPMI, permanent pacemaker implantation; SAVR, surgical aortic valve replacement; SMA, septal myocardial ablation; TAVR, transcatheter aortic valve replacement

336 patients treated with TAVR were included for model derivation, among whom 33 (9.8%) had new LBBB and 48 (14.3%) had PPMI. Major reasons for PPMI include CHB (37, 77.1%), HAVB (4, 8.3%), pre-existing atrial fibrillation with slow ventricular response (3, 6.3%), new incidence of LBBB (2, 4.2%), sinus arrest (1, 2.1%) and sick sinus syndrome (1, 2.1%) (Fig. 1). Dual-chamber pacemakers (VAT mode) were implanted for CHB and HAVB patients, single-chamber pacemaker (VVI mode) was implanted for patients with atrial fibrillation. For patients with sinus arrest and sick sinus syndrome, dual-chamber pacemakers (AAI mode) were implanted. For the two patients with new incidence of LBBB, dual-chamber pacemakers (VVI mode) were used. 38 patients had PPMI within 7 days of TAVR, while only 6 and 4 PPMIs were conducted between 8 to 30 days and beyond 30 days, respectively. Patient characteristics in the derivation data set are summarized in Table 1 and Additional file 1: Table S1. Compared to those without PPMI, patients requiring PPMI had similar demographics and disease history, higher prevalence of RBBB (18.75% vs 3.82%; *p* < 0.001) and larger PNA (5.46 ± 0.99 vs 5.16 ± 0.94; *p* = 0.048). PPMI group had longer PLOS than non-PPMI group (8.98 ± 3.64 vs 6.94 ± 3.08 days; *p* < 0.001).

**Table 1:** Patient characteristics in the derivation set

	Total (*n* = 336)	PPMI (*n* = 48)	No PPMI (*n* = 288)	*p* value
Baseline characteristics and medical history				
Age, yrs	75.69 ± 7.11	76.62 ± 6.87	75.53 ± 7.15	0.315
Male	142 (42.26%)	16 (33.33%)	126 (43.75%)	0.168
CAD	271 (80.65%)	37 (77.08%)	234 (81.25%)	0.527
Diabetes	84 (25%)	7 (14.58%)	77 (26.74%)	0.039
Hypertension	191 (56.85%)	27 (56.25%)	164 (56.94%)	0.929
History of stroke	18 (5.36%)	3 (6.25%)	15 (5.21%)	0.783
History of syncope	34 (10.12%)	6 (12.5%)	28 (9.72%)	0.590
Atrial fibrillation	68 (20.24%)	12 (25%)	56 (19.44%)	0.413
First degree AVB	17 (5.06%)	4 (8.33%)	13 (4.51%)	0.467
LBBB	7 (2.08%)	0 (0%)	7 (2.43%)	0.008
RBBB	20 (5.95%)	9 (18.75%)	11 (3.82%)	0.013
eGFR, ml/min	67.92 ± 22.32	65.88 ± 25.17	68.26 ± 21.83	0.539
Characteristics before TAVR				
Aortic annulus perimeter, mm	80.56 ± 7.96	81.5 ± 6.94	80.41 ± 8.12	0.328
Bicuspid aortic valve	187 (55.65%)	26 (54.17%)	161 (55.90%)	0.825
AVA, cm^2^	0.71 ± 0.22	0.77 ± 0.27	0.7 ± 0.20	0.084
LVEF, %	58 ± 11	58 ± 11	58 ± 11	0.950
TnT, ng/ml	0.07 ± 0.20	0.08 ± 0.24	0.07 ± 0.20	0.811
Transfemoral	325 (96.73%)	47 (97.92%)	278 (96.53%)	0.556
Characteristics after TAVR				
Self-expandable valve	329 (97.92%)	48 (100%)	281 (97.57%)	0.008
Oversizing, %	0.38 ± 7.86	1.37 ± 7.53	0.21 ± 7.91	0.315
PNA, cm^2^	5.21 ± 0.95	5.46 ± 0.99	5.16 ± 0.94	0.058
AVA, cm^2^	2.30 ± 0.61	2.33 ± 0.62	2.30 ± 0.61	0.701
Implantation depth, mm	5.22 ± 3.73	6.06 ± 3.56	5.08 ± 3.74	0.082
TnT, ng/ml	0.34 ± 0.45	0.44 ± 0.72	0.32 ± 0.39	0.267
PLOS, day(s)	7.23 ± 3.24	8.98 ± 3.64	6.94 ± 3.08	< 0.001

Values are mean ± SD or frequency (%). *p* values are obtained by two-sample *t*-test

AVA, aortic valve area; AVB¸ atrioventricular block; CAD, coronary artery disease; eGFR, estimated glomerular filtration rate; LVEF, left ventricular ejection fraction; LBBB, left bundle branch block; PLOS, postoperative length of stay; PNA, prosthetic nominal area; PPMI, permanent pacemaker implantation; RBBB, right bundle branch block; TnT, troponin-T

### Candidate predictor screening

**Fig. 2:**
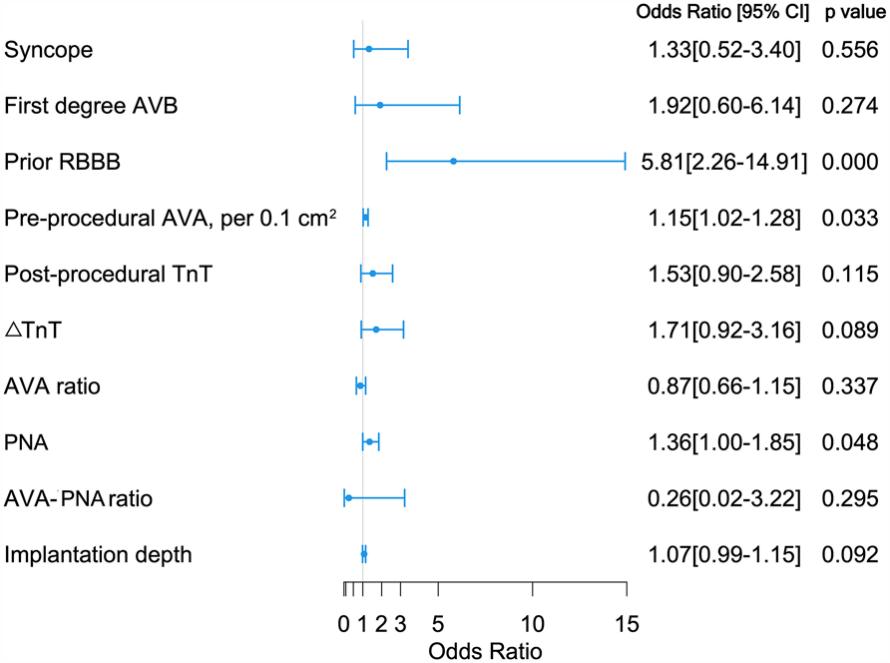
Univariate odds ratio (OR), 95% confidence interval and *p* value for the selected candidate predictors. AVA, aortic valve area; AVA ratio, ratio of post-procedural aortic valve area to pre-procedural area; AVA–PNA ratio, ratio of post-procedural aortic valve area to prosthetic nominal area; AVB, atrioventricular block; PNA, prosthetic nominal area; RBBB, right bundle branch block; TnT, troponin-T; △TnT, difference between post-procedural and pre-procedural troponin-T

Candidate predictors were selected based on univariate associations with PPMI as well as clinical significance. Figure 2 shows the univariate odds ratio and its corresponding 95% confidence interval for each candidate predictor. Among the 10 candidate predictors selected, syncope, first-degree AVB, RBBB, pre-procedural aortic valve area (pre-procedural AVA) were collected before TAVR, while post-procedural troponin-T, difference between post- and pre-procedural troponin-T (△TnT), ratio of post- to pre-procedural aortic valve area (AVA ratio), prosthetic nominal area (PNA), ratio of post-procedural aortic valve area to prosthetic nominal area (AVA–PNA ratio), implantation depth were collected after TAVR. Results from univariate analyses indicated that RBBB (OR = 5.81; *p* < 0.001) and large pre-procedural AVA (OR = 4.22; *p* = 0.033) were significant pre-procedural risk factors, while large PNA (OR = 1.36; *p* = 0.048) was significant post-procedural risk factors for PPMI.

### Pre- and post-procedural prediction models

**Fig. 3:**
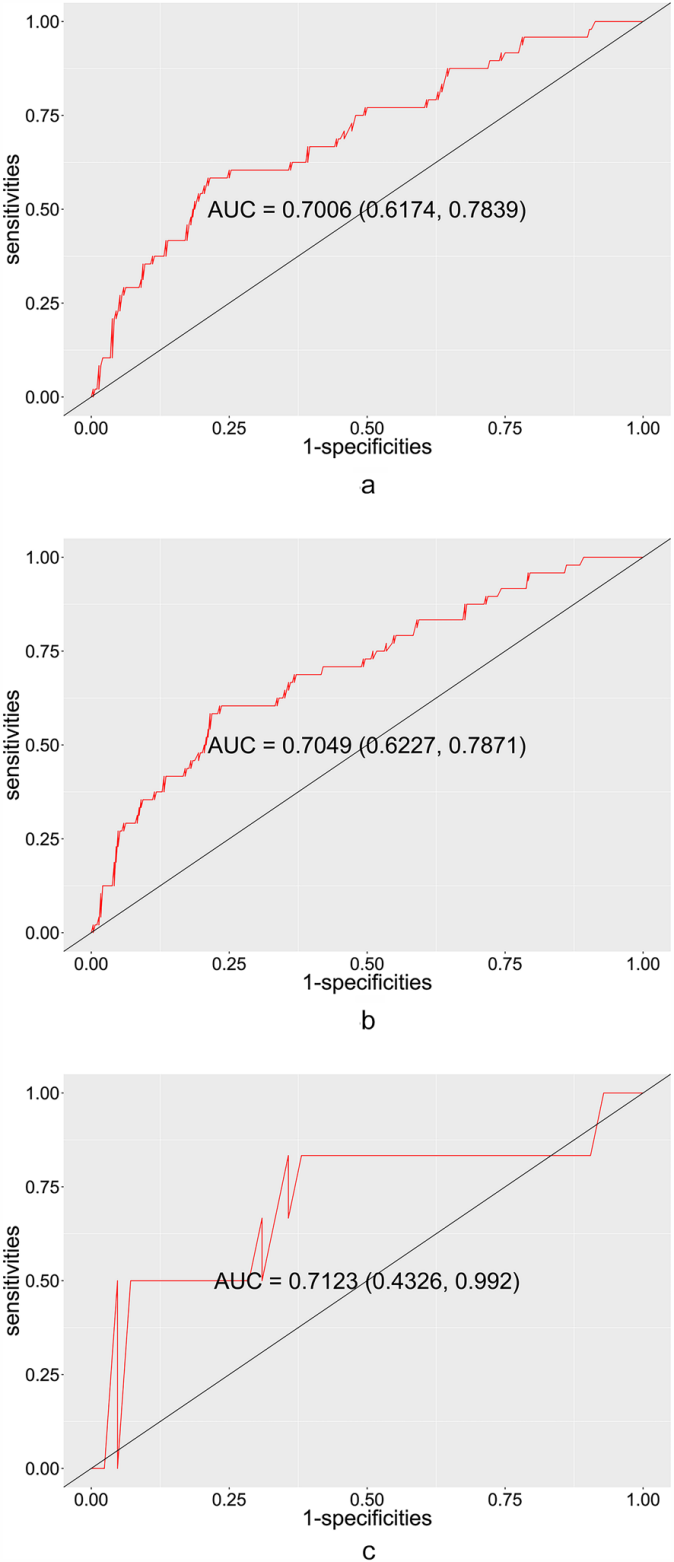
Receiver operating characteristics (ROC) curve in the derivation set (**a**), bootstrapped internal validation set (**b**), and external validation set (**c**). The AUROCs in the derivation, internal and external validation sets are 0.70 (95% CI 0.62–0.78), 0.70 (95% 0.62–0.79) and 0.71 (95% CI 0.43–0.99), respectively

Multivariate logistic regression models for the pre- and post-procedural prediction of PPMI are shown in Table 2. The multivariate odds ratios and corresponding 95% confidence intervals from the post-procedural model suggested that RBBB (OR = 10.46; *p* < 0.001), large pre-procedural AVA (OR = 1.41; *p* = 0.004) and large AVA ratio (OR = 1.72; *p* = 0.043) were independent risk factors for PPMI. Specifically, patients with RBBB were 10 times more likely to require PPMI after TAVR, and those with large pre-procedural AVA and large AVA ratio had increased risk of PPMI, adjusting for other factors in the model. Large AVA–PNA ratio was associated with reduced risk of PPMI, however, this effect did not achieve statistical significance (OR = 0.01; *p* = 0.052). Higher AUROC was obtained for the post-procedural prediction model (AUROC = 0.70, 95% CI 0.62–0.78, log likelihood = − 98.21, Fig. 3a) than the pre-procedural model (AUROC = 0.63, 95% CI 0.53–0.72, log likelihood = − 100.48, *p* = 0.012), indicating extra benefit of discriminative power by including post-procedural predictors. The precision–recall curves on the derivation set, internal validation set and external validation set are shown in Additional file 1: Figure S1 and the *F*-scores were 0.30, 0.31 and 0.30, respectively.

**Table 2:** Multivariate logistic regression models for pre- and post-procedural prediction of PPMI

	OR (95% CI)	*p* value
Pre-procedural prediction model		
Prior RBBB	9.36 (3.09, 29.23)	< 0.001**
Pre-procedural AVA, per 0.1 cm^2^	1.17 (1.00, 1.37)	0.042*
Post-procedural prediction model		
Prior RBBB	10.46 (3.40, 33.29)	< 0.001***
Pre-procedural AVA, per 0.1 cm^2^	1.41 (1.12, 1.79)	0.004**
AVA ratio	1.72 (1.00, 2.91)	0.043*
AVA–PNA ratio	0.01 (0.00, 0.92)	0.052

The multivariate regression models were obtained via forward and backward variable selection for the pre- and post-procedural prediction models (see “Methods”)

AVA ratio, ratio of post-procedural aortic valve area to pre-procedural area; AVA–PNA ratio, ratio of post-procedural aortic valve area to prosthetic nominal area; CI, confidence interval; OR, odds ratio; other abbreviations as in Table 1

Signif. codes: 0 ‘***’ 0.001 ‘**’ 0.01 ‘*’ 0.05 ‘.’ 0.1 ‘’ 1

### Risk score of the post-procedural prediction model

**Fig. 4:**
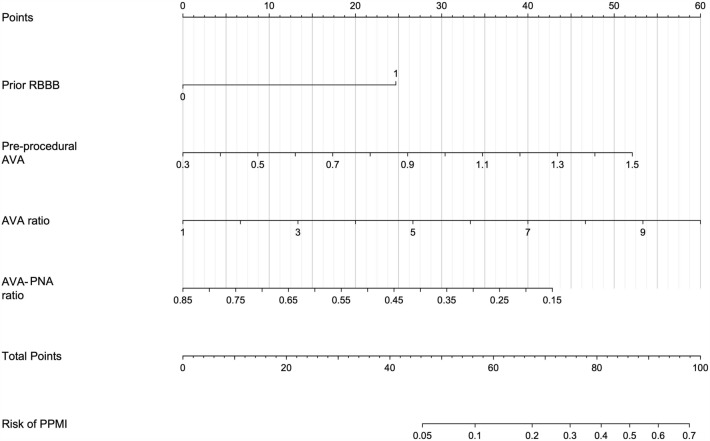
Nomogram showing the total risk score as well as the individual risk score associated with each predictor. Patients are classified into 4 risk groups according to their total risk scores: very low (0–40 points), low (40–60 points), moderate (60–80 points) and high (80–100 points). AVA, aortic valve area; AVA ratio, ratio of post-procedural aortic valve area to pre-procedural area; AVA–PNA ratio, ratio of post-procedural aortic valve area to prosthetic nominal area; PPMI, permanent pacemaker implantation; RBBB, right bundle branch block

**Fig. 5:**
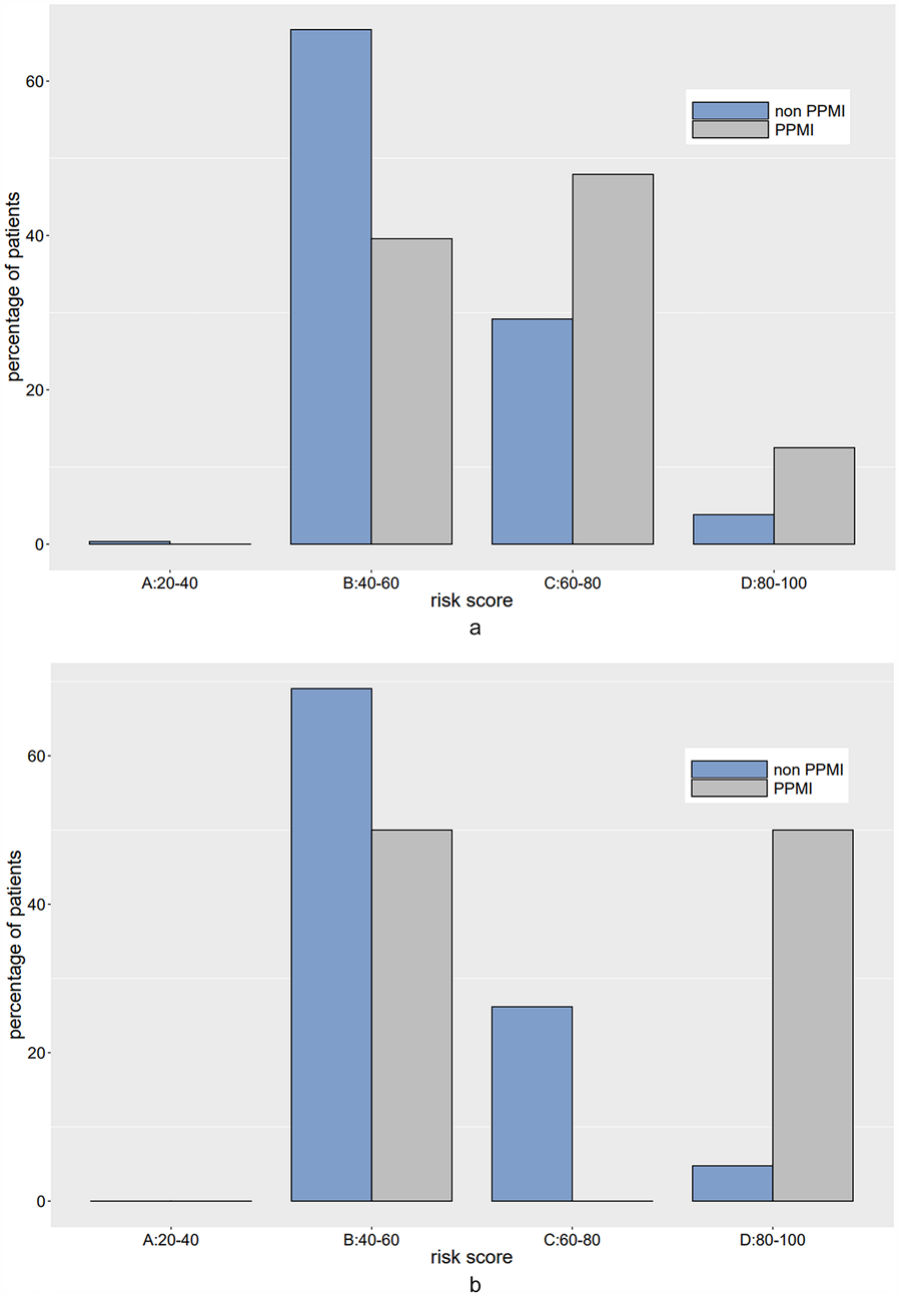
Distribution of the risk scores for people with and without PPMI in the derivation set (**a**) and external validation set (**b**). Risk scores are scaled from 0 to 100, with higher scores indicating higher risk of PPMI

Based on the post-procedural prediction model, we generated a nomogram (Fig. 4) showing the individual risk score associated with each predictor as well as the total risk score on the scale of 0 to 100, with higher scores indicating higher risk of PPMI. Patients were divided into 4 risk groups according to their risk scores: very low (0–40 points), low (40–60 points), moderate (60–80 points) and high (80–100 points), corresponding to risk probability of < 0.03, 0.03–0.13, 0.13–0.39 and > 0.39, respectively. For example, the total risk score for a post-TAVR patient who had RBBB, 0.4 cm^2^ pre-procedural AVA, 4 AVA ratio and 0.6 AVA–PNA ratio was approximately 25 + 4 + 20 + 15 = 64, and can be classified into the moderate risk group. Distribution of the total risk score for patients in the derivation set is provided in Fig. 5a. There are more PPMI cases in moderate and high risk groups relative to low and very low risk groups. The sensitivity, specificity, positive predictive value and negative predictive value for the low, medium and high score cutoffs are shown in Additional file 1: Table S2.

### Validation of the prediction model

We conducted both internal and external validation of the post-procedural prediction model. Comparison of patient characteristics in the derivation and external validation set is provided in Table 3. Compared to those in the derivation set, patients in the external validation set had lower incidence of cardiovascular disease history (62.5% vs 80.7%; *p* = 0.017), lower pre- (0.03 ± 0.03 vs 0.07 ± 0.20; *p* = 0.002) and post-procedural TnT (0.24 ± 0.16 vs 0.34 ± 0.45; *p* = 0.005), lower post-procedural AVA (2.07 ± 0.63 vs 2.30 ± 0.61; *p* = 0.022) and lower PNA (4.78 ± 0.97 vs 5.21 ± 0.95; *p* = 0.006). Despite these differences, they had similar baseline demographics and incidence of PPMI (12.50% vs 14.29%; *p* = 0.732) with those in the derivation set. The AUROC was 0.70 (95% CI 0.62–0.79) in the averaged bootstrap sample for the internal validation, and 0.71 (95% CI 0.43–0.99) in the external validation set. Similar pattern was observed for the distribution of the total risk scores in the external validation set, as shown in Fig. 5b.

**Table 3:** Comparison of patient characteristics in the derivation and external validation set

	Derivation (*n* = 336)	External validation (*n* = 48)	*p* value
Age, yrs	75.69 ± 7.11	73.79 ± 8.04	0.126
Male	142 (42.26%)	25 (52.08%)	0.211
CAD	271 (80.65%)	30 (62.5%)	0.017
RBBB	20 (5.95%)	3 (6.25%)	0.937
Pre-procedural TnT, ng/ml	0.07 ± 0.20	0.03 ± 0.03	0.002
Post-procedural TnT, ng/ml	0.34 ± 0.45	0.24 ± 0.16	0.005
Pre-procedural AVA, cm^2^	0.71 ± 0.22	0.72 ± 0.32	0.885
Post-procedural AVA, cm^2^	2.30 ± 0.61	2.07 ± 0.63	0.022
PNA, cm^2^	5.21 ± 0.95	4.78 ± 0.97	0.006
Implantation depth, mm	5.22 ± 3.73	5.46 ± 3.58	0.665
New-onset LBBB	9.82%	8.33%	0.948
Incidence of PPMI	14.29%	12.50%	0.732
PLOS, day(s)	7.23 ± 3.24	7.40 ± 5.92	0.230

Values are mean ± SD or frequency (%). *p* values are obtained by two-sample *t* test or Chi-square test. Abbreviations as in Table 1

### Time-to-PPMI analysis

Predictors that potentially influence patients’ time-to-PPMI were investigated using Cox proportional hazard model and results are provided in Table 4. In brief, patients with prior RBBB (HR = 5.07; *p* < 0.001), large pre-procedural AVA (HR = 1.33; *p* = 0.001), small AVA–PNA ratio (HR = 0.02; *p* = 0.039) and large △TnT (HR = 1.72; *p* = 0.017) were more likely to have shorter time from TAVR to PPMI, adjusting for other factors in the model. Large AVA ratio was a risk factor for time-to-PPMI, but was not statistically significant at 0.05 level (HR = 1.50; *p* = 0.053). Kaplan–Meier curves (Additional file 1: Figure S2) showed that most PPMI cases occurred within 7 days after TAVR, and patients with prior RBBB had significantly higher risks of PPMI at all times after TAVR (*p* < 0.001). A nomogram showing the risk score associated with time-to-PPMI is provided in Additional file 1: Figure S3.

**Table 4:** Cox proportional hazard model for the time-to-PPMI

	Hazard ratio (95% CI)	*p* value
Prior RBBB	5.07 (2.44, 10.56)	< 0.001***
Pre-procedural AVA, per 0.1 cm^2^	1.33 (1.12, 1.59)	0.001**
AVA ratio	1.50 (1.00, 2.27)	0.053
AVA–PNA ratio	0.02 (0.0007, 0.82)	0.039*
△TnT	1.72 (1.10, 2.67)	0.017*

△TnT: difference between post-procedural and pre-procedural troponin-T; other abbreviations as in Tables 1 and 2

Signif. codes: 0 ‘***’ 0.001 ‘**’ 0.01 ‘*’ 0.05 ‘.’ 0.1 ‘’ 1

## Discussion

PPMI due to conduction block remains one of the major complications after TAVR, and there has been limited research on effective prediction models involving both pre- and post-procedural characteristics. In this study, we developed a post-procedural prediction model for PPMI via multivariate logistic regression and identified RBBB, pre-procedural AVA, post- to pre-procedural AVA ratio, and post-procedural AVA to PNA ratio as independent predictors for PPMI. Among them, having prior RBBB, large pre-procedural AVA and large post- to pre-procedural AVA ratio are risk factors, while large post-procedural AVA to PNA ratio is protective for PPMI. To our knowledge, AVA ratio and AVA–PNA ratio are identified as independent predictors for the first time and these two factors may related to the mechanical stress of the prosthesis. Total risk score can be calculated by adding up individual risk score associated with each predictor according to the post-procedural prediction model, and patients can then be classified into different risk groups based on their total risk score. The model has been validated internally via bootstrap and externally via an independent cohort of patients from the same hospital. Studies showed that incidence of delayed PPMI is increasing [25]. Therefore, we investigated the effects of potential predictors on how soon PPMI could occur quantitatively as the secondary analysis.

RBBB has been reported as a risk factor for PPMI in many previous studies [9, 10, 13] and was also confirmed in our analysis. The high incidence of new-onset LBBB after TAVR [5] could explain why prior RBBB strongly associated with CHB or HAVB.

The effect of pre-procedural AVA on PPMI is likely related to the buffering force of the prosthetic valve holder to surrounding tissue due to the stenosis valve. Since the atrioventricular conduction system is anatomically close to the subaortic valve structure, the risk of pressure damage to the conduction system during valve implantation is difficult to avoid [26]. Senile aortic stenosis is often associated with severe calcification, and a reduction in valve area often means an increase in surrounding calcified tissue, which provides good support for the implanted valve and may reduce the direct pressure of the prosthetic valve holder on the subvalve tissue. The contact point between bicuspid aortic valve (BAV) and prosthetic valve holder may be closer to the axis and further away from membranous septum, therefore, pre-procedural AVA is more likely to affect the Chinese population with a higher prevalence of BAV stenosis. This may also explain why pre-procedural AVA was not found to be associated with PPMI among European and American population [11].

Post- to pre-procedural AVA ratio reflects the extent to which tissue is squeezed between the prosthetic valve holder and the aortic wall after prosthetic implantation, with higher ratio indicating more squeezing and higher pressure of the prosthetic valve holder on the aortic wall and left ventricular outflow tract. High prosthetic pressure on surrounding tissues, especially the vulnerable areas where the His bundle and the left bundle branches locate, is a risk factor for PPMI [27, 28]. Therefore, large post- to pre-procedural AVA ratio increases the risk of damage to the conduction system and PPMI after TAVR. Although AVA ratio and AVA–PNA ratio did not achieve statistical significance (AVA ratio: HR = 1.50, *p* = 0.053; AVA–PNA ratio: OR = 0.01; *p* = 0.052) at the 0.05 significance level, we would still consider them as important predictors for the time-to-PPMI and PPMI, given the relatively small sample size in the study. AVA–PNA ratio is an estimate of the relative expansion of the prosthetic valve holder, with smaller AVA–PNA ratio indicating more compression (i.e., less expansion) of the prosthetic on surrounding tissues. In vitro experiments also confirmed that the radial expansion of the prosthetic valve was approximately linearly related to its compression [29]. However, estimation of the valve pressure and tissue compression was mostly done using finite element analysis (FEA) in the simulation setting, and is difficult to generalize to clinical settings due to technical limitation. In this study, we used a simple and more assessable approach by calculating the post-procedural AVA to PNA ratio as an alternative measure of tissue compression, and confirmed high prosthetic pressure as the risk factor for PPMI after TAVR [27, 28].

AVA ratio and AVA–PNA ratio were both used to evaluate the mechanical stress. AVA ratio estimates the expansion of the aortic valve tissue pushed by prosthesis, while AVA–PNA ratio reflects the deformation of prosthesis under compression. The compressed prosthesis generated radial force due to continuous expansion of the memory metal, while the expansion level of the aortic valve tissue around the prosthesis reflects the response of the surrounding tissue to the force, which shows the stiffness and compliance of the tissue. The occurrence of PPMI after TAVR is closely related to the pressure of the prosthesis on the membranous septum, and the pressure degree is closely related to the compression of the valve frame. However, for patients with the same annulus size and the same prosthesis, the actual compression ratio of the prosthesis may not be the same due to different situation of valve leaf calcification, adhesion and fusion. Therefore, AVA–PNA ratio is likely to better reflect the above situation. This would explain why AVA–PNR ratio, rather than oversizing rate, was significantly different between PPMI and non-PPMI patients.

Previous studies have shown that myocardial injury and elevated troponin after TAVR were associated with increased risk of PPMI [30, 31]. In this study, we assessed the elevation of troponin using the difference between post- and pre-procedural troponin-T. To better reflect the myocardial injury specifically caused by TAVR, we excluded patients undergoing concurrent PCI, SMA, and electrical cardioversion. However, the post-relative to pre-procedural difference of TnT was not statistically associated with binary PPMI endpoint due to small sample size. In the secondary analysis involving time-to-PPMI, the post-relative to pre-procedural difference of TnT was identified as a significant risk factor and patients with higher elevated troponin were more likely to have PPMI sooner, suggesting that early occurrence of PPMI might be caused by acute myocardial injury due to the value pressure of TAVR. However, the specificity of troponin is limited due to the simultaneous presence of coronary embolism caused by small emboli detachment during valve release [32], and the effect of intra-procedural tachyarrhythmia.

Evidence from previous studies showed that PPMI can occur beyond 30 days after TAVR [33]. The medical records of patients with PPMI beyond 30 days after TAVR in the derivation set showed that most of these patients experienced amaurosis or syncope within 30 days after TAVR, but did not seek appropriate medical treatment in time. The latest PPMI in the derivation set was observed on day 58 after TAVR due to symptomatic atrial fibrillation with slow ventricular response. Therefore, we decided to focus on the PPMI cases within 60 days after TAVR in the primary analysis. In the secondary analysis, we investigated potential factors that might influence patients’ time-to-PPMI and found that in addition to acute myocardial injury, greater valve pressure as indicated by large pre-procedural AVA, post- to pre-procedural AVA ratio and small post-procedural AVA to PNA ratio were associated with sooner PPMI.

Delayed high-degree atrioventricular block after TAVR is increasing in recent years, which may due to the early-discharge practices and surveillance strategies after discharge [34]. This may cause syncope and fall out of the hospital, which may fatal to the already elderly patients. The prediction of delayed PPMI occurrence is however difficult and the related risk factor is still unknown. Current thinking suggests that the persistent oppression caused by the self-expandable valves maybe one of the main reasons. The factors derived from our predictive model may reflect the oppression stress and showed relevance with the timing of PPMI which may useful for future research.

The prediction model achieved good discriminative power in both the internal and external validation. For example, Shivamurthy et al. included transfemoral approach, LBBB without bradycardia, sinus bradycardia without LBBB, RBBB, LBBB with sinus bradycardia, and second-degree AVB in a prediction model with AUROC 0.674 [19, 35]. In another prediction model by Tsushima et al., hypertension, first-degree AVB, self-expanding valve use and RBBB were selected as potential predictors for PPMI and the AUROC was 0.693 [18]. We verified these two models and obtained an AUROC of 0.57 and 0.58 using patient data in the derivation set, respectively (Additional file 1: Figure S4), which suggests that existing prediction models might not be appropriate for the Chinese population.

Previous studies have suggested other predictors for PPMI, such as the distribution and sizing of aortic valve calcification and membranous septum length [11]. However, these predictors cannot be easily obtained in clinical settings since they require specific imaging protocols and experienced technicians/physicians for image interpretations. Therefore, we chose not to include them in the predictive models in this investigation. Artificial intelligence is growing rapidly in recent years. The previously reported machine learning-based prediction models demonstrated significantly high predictive accuracy [36, 37]. Due to the restricted data volume, machine learning is not applicable to our study yet. By using this method, we need to screen the input variables first, because in restrict to the computer calculation speed, we cannot put in the whole database. Our study may give some insights about the variables choosing in future research, especially about the delayed PPMI after TAVR. The majority of the existing prediction models for PPMI included only the pre-procedural predictors and obtained patient susceptibility before undergoing TAVR. The prediction model developed in our study combined both pre- and post-procedural characteristics and incorporated patient susceptibility into the total risk score after TAVR, and is thus more clinically meaningful for patients with high risk of PPMI.

### Study limitations

First, the sample size in our study is small. According to the 10 event-per-variable (EPV) rule-of-thumb that the number of predictors in the model should not exceed one-tenth of the total number of events, we included only 4 predictors in the post-procedural prediction model. As a result, variables that were associated with PPMI in previous findings, such as history of syncope [17], hypertension [18], valve type [18] and oversizing rate [17], were not included due to insufficient power. Second, the majority of patients in our study were implanted with self-expanding valve. Therefore, the prediction model might not be generalizable to patients with balloon-expanding valve. Third, patients in the derivation set and external validation set had notable differences in terms of cardiovascular disease history, TnT and post-procedural AVA, which might affect results for external validation. Fourth, this is a single-center study and might not generalize to patients from other medical centers, although quite a bit of the TAVR within mainland China were conducted in our department. Fifth, our study is retrospective in design without prospective validation, and might be subject to selection bias. However, predictors identified in our post-procedural prediction model are all physiologically and anatomically reasonable. Sixth, the electrocardiogram after TAVR in our center is made in paper version beside the bed, electronic information is lacking. And we found many of the thermal drawings of 4–5 years ago can’t be seen clearly right now, so we can’t use them. Thus, we can’t analyze factors like the prolongation of QRS and PR after TAVR. But LBBB, CHB, HAVB and other cases requiring PPMI have been recorded in the disease course in time, this is the source of our data. Seventh, for patients with sinus rest and sick sinus syndrome, pacemakers are usually implanted before TAVR, and such patients are excluded from this study. But the two patients in our study showed normal electrocardiogram before TAVR, so it’s difficult to determine if this is related to TAVR or if it just wasn't detected by a regular electrocardiogram before TAVR. This may have some impact on the results.

## Conclusions

We showed that prior RBBB, large pre-procedural AVA, large post- to pre-procedural AVA and small post-procedural AVA to PNA ratio increased the risk of PPMI for patients undergone TAVR. Based on the prediction model, we established and validated a risk score system for PPMI on the scale of 0 to 100, which is easily applicable in clinical setting regarding the Chinese population.

## Supplementary Information

**Additional file 1: Figure S1.** The precision-recall curve on the derivation set (a), internal validation set (b) and external validation set (c). **Figure S2.** The Kaplan–Meier survival curves for patients in the derivation set (a) and stratified for patients with and without prior RBBB (b). **Figure S3.** Nomogram for the risk score system of time-to-PPMI. **Table S1.** Supplement of patient characteristics in the derivation set. **Table S2.** Sensitivity, specificity, PPV and NPV based on low, medium, and high score cut offs. **Table S3.** The missing proportions.

## Data Availability

The datasets generated and analyzed during the current study are not publicly available due to the violation of patient privacy and the absence of informed consent for online raw data. Still, they are available from the corresponding author upon reasonable request.

## Abbreviations

AVA ratio: Ratio of post-procedural aortic valve area to pre-procedural area
AVA–PNA ratio: Ratio of post-procedural aortic valve area to prosthetic nominal area
△TnT: Difference between post-procedural and pre-procedural troponin-T
