# Supplementary material for: A prediction model for permanent pacemaker implantation after transcatheter aortic valve replacement
Source: Eur J Med Res. 2023 Jul 29;28:262. doi: 10.1186/s40001-023-01237-w (PMC10387194; doi:10.1186/s40001-023-01237-w)
Supplement: Supplementary file 1 — Additional file 1: Figure S1. The precision-recall curve on the derivation set (a), internal validation set (b) and external validation set (c). Figure S2. The Kaplan–Meier survival curves for patients in the derivation set (a) and stratified for patients with and without prior RBBB (b). Figure S3. Nomogram for the risk score system of time-to-PPMI. Table S1. Supplement of patient characteristics in the derivation set. Table S2. Sensitivity, specificity, PPV and NPV based on low, medium, and high score cut offs. Table S3. The missing proportions. [file 40001_2023_1237_MOESM1_ESM.docx]

**Additional Figure Titles and Legends**

Figure S1. The precision-recall curve on the derivation set (a), internal validation set (b) and external validation set (c).

**
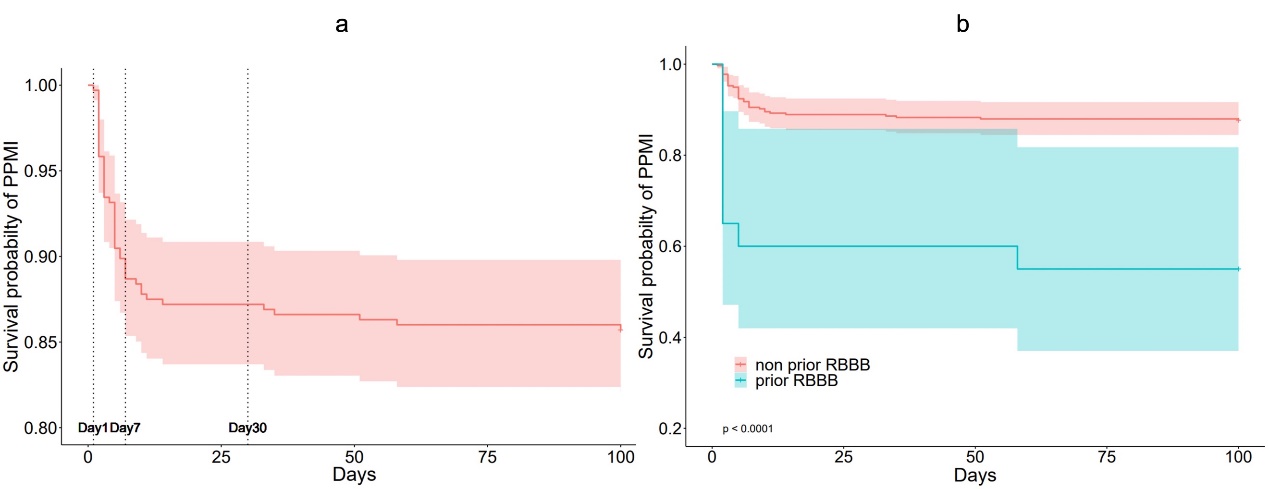
**

Figure S2. The Kaplan-Meier survival curves for patients in the derivation set (a) and stratified for patients with and without prior RBBB (b). PPMI = permanent pacemaker implantation; RBBB = right bundle branch block.

Figure S3. Nomogram for the risk score system of time-to-PPMI. AVA = aortic valve area; AVA ratio = ratio of post-procedural aortic valve area to pre-procedural area; AVA-PNA ratio = ratio of post-procedural aortic valve area to prosthetic nominal area; RBBB = right bundle branch block; △TnT = difference between post-procedural and pre-procedural troponin-T.


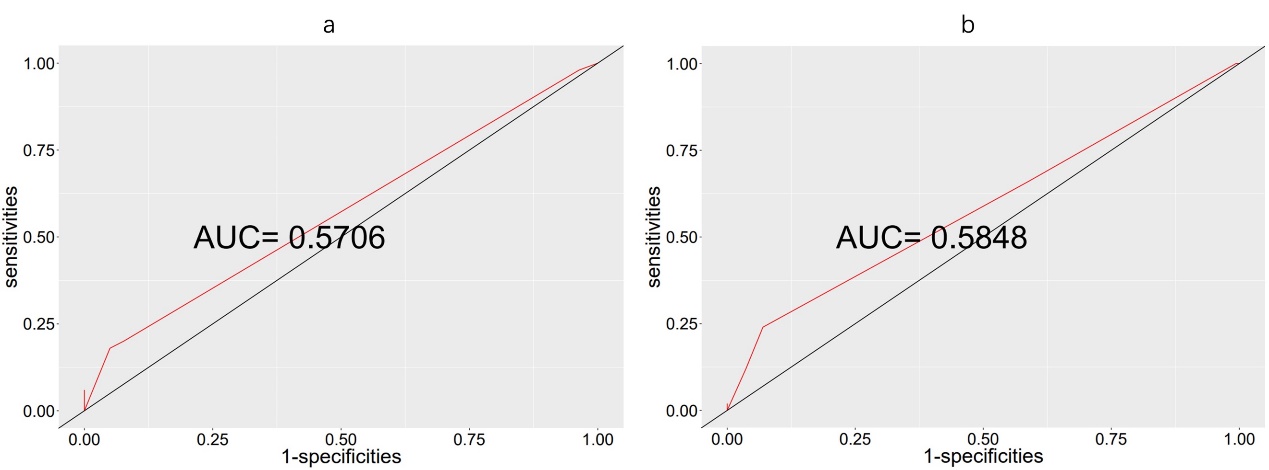


Figure S4. The derivation set evaluated by Shivamurthy et al.’s (a) and Tsushima et al.’s (b) risk prediction model. AUC = area under the receiver operating characteristic curve.

Table S1. Supplement of patient characteristics in the derivation set.

|  | Total  (n=336) | PPMI  (n=48) | no PPMI  (n=288) | p value |
| --- | --- | --- | --- | --- |
| **Baseline characteristics and medical history** | | | | |
| History of MI | 12(3.57%) | 1(2.08%) | 11(3.82%) | 0.555 |
| History of PCI | 45(13.39%) | 4(8.33%) | 41(14.24%) | 0.269 |
| History of CABG | 5(1.49%) | 2(4.17%) | 3(1.04%) | 0.126 |
| Smoker | 45(13.39%) | 8(16.67%) | 37(12.85%) | 0.473 |
| COPD | 39(11.61%) | 6(12.5%) | 33(11.46%) | 0.835 |
| **Characteristics before implantation** | | | | |
| Pre-procedural MPGE, mmHg | 46.29 ± 17.25 | 44.17 ± 14.31 | 46.65 ± 17.69 | 0.356 |
| Pre-procedural PPGE, mmHg | 75.85 ± 25.88 | 72.48 ±23.11 | 76.42 ± 26.31 | 0.329 |
| MVPBIC, mmHg | 179.40 ± 37.02 | 176.04 ± 40.14 | 179.95 ± 36.51 | 0.497 |
| MAPBIC, mmHg | 107.99 ± 20.78 | 106.06 ± 20.96 | 108.31 ± 20.77 | 0.487 |
| Balloon predilation | 328(97.62%) | 47(97.92%) | 281(97.57%) | 0.884 |
| **Characteristics after implantation** | | | | |
| Post-procedural MPGE, mmHg | 8.04 ± 4.28 | 8.00 ± 4.31 | 8.04 ± 4.28 | 0.950 |
| Post-procedural PPGE, mmHg | 15.23 ± 7.65 | 15.08 ± 7.29 | 15.25 ± 7.72 | 0.889 |
| MVPAIC, mmHg | 129.91 ± 21.38 | 128.44 ± 23.45 | 130.15 ± 21.05 | 0.606 |
| MAPAIC, mmHg | 122.23 ± 21.67 | 121.02 ± 23.50 | 122.43 ± 21.39 | 0.677 |
| Balloon postdilation | 103(30.65%) | 13(27.08%) | 90(31.25%) | 0.563 |

Values are mean ± SD or frequency (%).

CABG = coronary artery bypass grafting; COPD = chronic obstructive pulmonary disease; MI = myocardial infarction; MPGE = mean pressure gradient by echocardiography; MVPBIC = maximum ventricular pressure before implantation via catheter; MAPBIC = maximum aortic pressure before implantation via catheter; MVPAIC = maximum ventricular pressure after implantation via catheter; MAPAIC = maximum aortic pressure after implantation via catheter; PPMI = permanent pacemaker implantation; PCI = percutaneous coronary intervention; PPGE = peak pressure gradient by echocardiography.

Table S2. Sensitivity, specificity, PPV and NPV based on low, medium, and high score cut offs

| Derivation set | | | | |
| --- | --- | --- | --- | --- |
|  | sensitivity | specificity | PPV | NPV |
| Cutoff: Positive rate in derivation set | 0.521 | 0.806 | 0.309 | 0.910 |
| Cutoff: Rate between low and medium | 0.083 | 0.986 | 0.5 | 0.866 |
| Cutoff: Rate between medium and high | 0 | 1 | - | 0.857 |
| External validation set | | | | |
|  | sensitivity | specificity | PPV | NPV |
| Cutoff: Positive rate in derivation set | 0.5 | 0.810 | 0.273 | 0.919 |
| Cutoff: Rate between low and medium | 0 | 0.952 | 0 | 0.870 |
| Cutoff: Rate between medium and high | 0 | 1 | - | 0.875 |

NPV = negative predictive value; PPV = positive predictive value.

Table S3. The missing proportions

| Predictors | Missing | Missing proportions |
| --- | --- | --- |
| eGFR | 1 | 0.30% |
| LVEF | 4 | 1.19% |
| Pre-procedural AVA | 12 | 3.57% |
| Post-procedural AVA | 13 | 3.87% |
| Post-procedural PPGE | 1 | 0.30% |
| Post-procedural MPGE | 2 | 0.60% |
| AVA ratio | 19 | 5.65% |
| MVPBIC | 11 | 3.27% |
| MAPBIC | 11 | 3.27% |
| MVPAIC | 16 | 4.76% |
| MAPAIC | 16 | 4.76% |
| AVA-PNA ratio | 13 | 3.87% |
| Implantation depth | 50 | 14.88% |

AVA = aortic valve area; AVA ratio = ratio of post-procedural aortic valve area to pre-procedural area; AVA-PNA ratio = ratio of post-procedural aortic valve area to prosthetic nominal area; eGFR = estimated glomerular filtration rate; LVEF = left ventricular ejection fraction; MPGE = mean pressure gradient by echocardiography; MVPBIC = maximum ventricular pressure before implantation via catheter; MAPBIC = maximum aortic pressure before implantation via catheter; MVPAIC = maximum ventricular pressure after implantation via catheter; MAPAIC = maximum aortic pressure after implantation via catheter; PPGE = peak pressure gradient by echocardiography.
